# A Rare EEG Finding of Eye Closure Sensitivity in a Child with Genetic Generalized Epilepsy: A Case Report

**DOI:** 10.3390/neurolint18070122

**Published:** 2026-06-24

**Authors:** Rayya Ali S. Almarwani, Anas Muslih B. Alkalbi, Juan Toro Perez

**Affiliations:** 1 Department of Pediatrics, King Faisal Specialist Hospital and Research Centre, Madinah 11211, Saudi Arabia; 2 Department of Neurophysiology, King Faisal Specialist Hospital and Research Centre, Madinah 11211, Saudi Arabia; alkalbaianas@gmail.com; 3 Department of Pediatric Neurology, Children’s Hospital of Eastern Ontario, University of Ottawa, Ottawa, ON K1H 8L1, Canada; jtoroperez@cheo.on.ca

**Keywords:** eye-closure sensitivity, genetic generalized epilepsy, tonic–clonic seizures, electroencephalography (EEG)

## Abstract

**Background**: Eye-closure sensitivity (ECS) is a rare reflex epilepsy phenomenon characterized by epileptiform discharges on electroencephalogram (EEG), triggered by eye closure. It has been reported in all genetic generalized epilepsies (GGEs), particularly in adolescents and adults. However, pediatric cases remain uncommon in the literature. **Case Presentation**: We report a 10-year-old previously healthy girl who presented with recurrent generalized tonic–clonic seizures beginning at age nine. Seizures occurred every few months without identifiable triggers, lasting 1–2 min with complete loss of consciousness, limb stiffening, rhythmic jerking, and upward eye deviation. Her developmental history was unremarkable, with no family history of epilepsy or febrile seizures. Neurological examination was normal. Initial EEG revealed intermittent generalized spike-and-wave and polyspike-and-wave discharges at 3 Hz (range 2–4 Hz), triggered by eye closure, consistent with ECS. These discharges occurred immediately following both spontaneous and instructed eye closure, were more prominent during drowsiness, and resolved upon eye opening. The patient remained alert during these subclinical events. No photosensitivity or hyperventilation response was observed. Brain magnetic resonance imaging was normal. The patient’s electroclinical findings were most consistent with a GGE phenotype with prominent ECS. She was treated with levetiracetam and has remained seizure-free for approximately 1.5 years to date. **Conclusions**: This case demonstrates that ECS can present in pediatric patients with GGE primarily manifested as generalized tonic–clonic seizures. EEG evaluation should include repeated eye-open/close maneuvers to unmask ECS, particularly in children with suspected generalized seizures.

## 1. Introduction

Genetic generalized epilepsy (GGE) encompasses a family of idiopathic epilepsy syndromes (e.g., childhood absence epilepsy, juvenile absence epilepsy, juvenile myoclonic epilepsy) presumed to have a genetic basis. In children, GGE is a major epilepsy category, with recent studies estimating that it constitutes about 23–35% of all epilepsy syndromes [1]. These epilepsies classically present with diffuse, bilateral synchronous spike-wave or polyspike-wave discharges on electroencephalogram (EEG). Common EEG activation procedures include hyperventilation for absence seizures and intermittent photic stimulation for photosensitive forms like Jeavons syndrome [2].

Eye closure sensitivity (ECS) is a distinctive form of reflex epilepsy characterized by the non-incidental appearance of transient epileptiform discharges on EEG, typically high-amplitude generalized spike-wave or polyspike-wave complexes at frequencies of 3–6 Hz, within 1–3 s following eye closure. These discharges last 1–4 s without persistence throughout the entire closure period [3]. Unlike photosensitivity, which responds to intermittent light stimulation, ECS is specifically triggered by the act of eye closure itself and represents a unique visual reflex phenomenon that warrants careful clinical recognition [4]. In addition, lacking clinical myoclonia makes ECS different from epilepsy with eyelid myoclonia (EEM), a GGE characterized by generalized polyspikes and spike-wave discharges on eye closure, brief, repetitive myoclonic jerks of the eyelids, and frequently associated absence seizures [5].

ECS occurs predominantly in females and typically manifests in childhood, with seizures starting on average between 6 and 12 years of age. This phenomenon has been reported in idiopathic generalized syndromes, including childhood or juvenile absence epilepsy, juvenile myoclonic epilepsy (JME), EEM, also known as Jeavons syndrome, and focal occipital epilepsies [6,7].

The pathophysiology of ECS is not fully understood, but it likely involves abnormal modulation of occipital alpha rhythms and thalamo-cortical excitability at eye closure [8]. In routine practice, ECS is often missed because standard EEG recordings do not emphasize repeated eye-closure maneuvers. Detecting ECS requires deliberate testing as patients should be asked to repeatedly open and close their eyes in a well-lit room, and standard activation techniques (hyperventilation and intermittent photic stimulation) should also be performed [9].

This case report presents a 10-year-old girl with generalized tonic–clonic seizures and prominent ECS on EEG, contributing to the limited pediatric literature on this rare phenomenon. The objectives of this report are to highlight the importance of recognizing ECS as a distinct reflex epilepsy mechanism and to understand its implications for syndrome classification, treatment selection, and long-term management in pediatric patients.

## 2. Case Presentation

A previously healthy 10-year-old girl was referred to our pediatric neurology clinic for evaluation of recurrent, unprovoked seizures. According to her parents, preictally, the seizures began at the age of nine, without any preceding illness or identifiable triggers. Ictally, the episodes were described as sudden, symmetrical (or occasionally asymmetrical) stiffness of the four limbs, loss of consciousness, often accompanied by upward deviation of the eyes. This phase was followed by rhythmic jerking movements, consistent with generalized tonic–clonic seizures. Each seizure lasted approximately 1–2 min. Postictally, the patient had a period marked by confusion and somnolence lasting several minutes. Her parents denied any preceding aura, vomiting, urinary or fecal incontinence, tongue biting, or postictal focal weakness. Seizure frequency was low, occurring approximately once every few months, and no clear provoking factors such as sleep deprivation, illness, fever, or emotional stress were identified.

Her past medical history was unremarkable. She was born full-term via spontaneous vaginal delivery following an uncomplicated pregnancy, with no perinatal issues or need for neonatal intensive care. Growth and development were age-appropriate across all domains, and she had no academic or behavioral concerns. There was no history of head trauma, central nervous system infections, or metabolic derangements. Family history was negative for epilepsy, febrile seizures, or other neurological or developmental disorders.

During physical examination, the patient appeared well, alert, cooperative, and developmentally appropriate for her age. Her growth parameters were within normal limits. Her neurological examination revealed no abnormalities. There were no dysmorphic features or neurocutaneous markers such as café-au-lait spots, hypopigmented macules, or vascular lesions. No focal neurological deficits were identified, and there were no signs suggesting a structural, metabolic, or syndromic cause of epilepsy.

An initial routine interictal video EEG was performed using the standard 10–20 electrode placement system. The recording included repeated spontaneous and instructed eye-opening and eye-closing maneuvers in a well-lit room, as well as hyperventilation for 5 min and intermittent photic stimulation. These activation procedures were used to increase the likelihood of unmasking reflex epileptiform activity. The technical quality of the recording was satisfactory, and no additional signal-processing pipeline beyond standard clinical EEG interpretation was required. The posterior dominant rhythm was 9 Hz, appropriate for age, with a rhythmic, symmetric, and reactive alpha activity primarily seen over the occipital regions. A mild anterior–posterior gradient was also noted. The patient was drowsy during the recording but did not enter stage II sleep.

**Figure 1:**
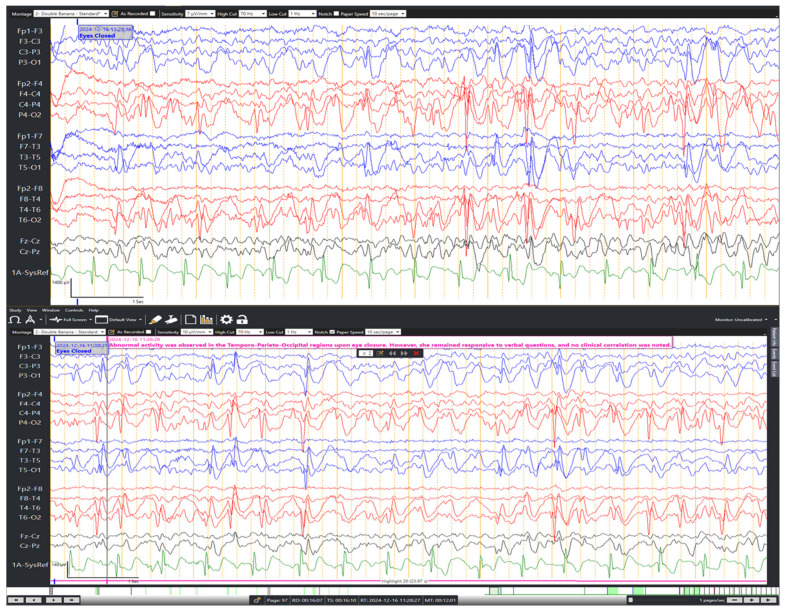
Bipolar and circumferential EEG montages showing generalized spike-and-wave and polyspike-and-wave discharges with posterior predominance over the temporo-occipital region, predominantly at 3 Hz. These discharges were triggered by eye closure.

**Figure 2:**
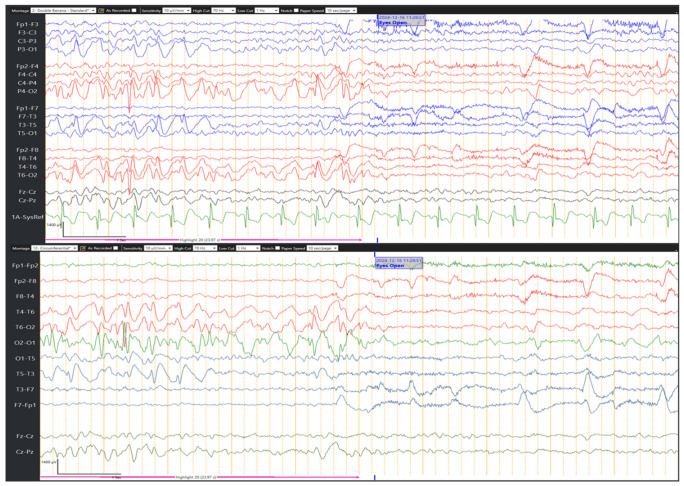
Bipolar and circumferential EEG montages showing that generalized discharges resolved immediately upon eye opening.

On bipolar and circumferential EEG montages, intermittent bursts of generalized spike-and-wave and polyspike-and-wave discharges were observed, predominantly at 3 Hz with occasional slowing to 1–2 Hz. The discharges showed posterior predominance over the temporo-occipital region and were predominantly triggered by eye closure (Figure 1). These discharges occurred immediately following both spontaneous and instructed eye closure and were more prominent during drowsiness. Each episode of discharges lasted several seconds and resolved promptly upon eye opening (Figure 2). However, the patient remained fully alert and responsive during the EEG events, with no associated clinical changes, such as behavioral arrest, motor automatism, or impaired awareness. No photic sensitivity was elicited during intermittent photic stimulation. Additionally, hyperventilation for 5 min did not provoke any abnormalities. ECG monitoring throughout the recording remained normal, including during the epileptiform events.

**Figure 3:**
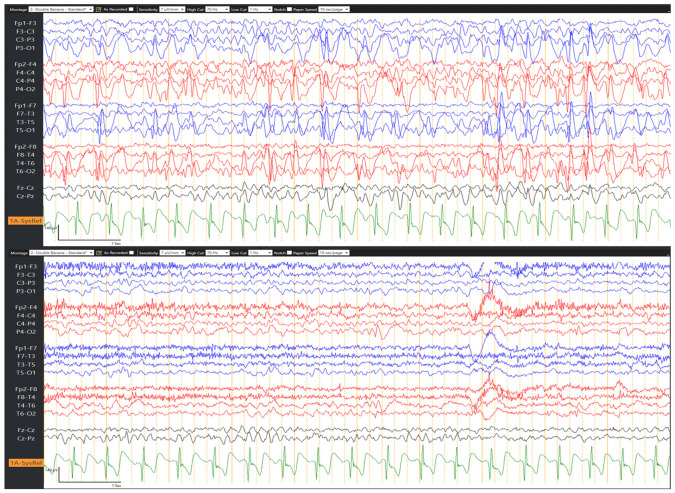
Follow-up bipolar EEG montages showing bilateral focal epileptiform discharges with predominantly 3 Hz spike-and-wave morphology. The discharges were maximal over the temporo-occipital areas, triggered by eye closure (**top**), and improved on eye opening (**bottom**).

A follow-up EEG was performed after 4 months, which demonstrated bilateral focal epileptiform discharges, predominantly 3 Hz spike-and-wave in morphology, maximal over the temporo-occipital areas (Figure 3). These discharges were more sustained during drowsiness and following eye closure, reinforcing the possibility of ECS epileptiform activity. Sleep was not obtained during either EEG. Artifacts were considered unlikely during the recordings because the discharges were reproducibly time-locked to eye closure, resolved on eye opening, and occurred while the patient remained alert and responsive without associated clinical change.

Brain magnetic resonance imaging (MRI) was performed to exclude structural abnormalities. It demonstrated normal brain parenchyma with no evidence of cortical malformations, hippocampal sclerosis, or other structural lesions. White matter appeared normal, and there were no signs of prior injury or developmental abnormalities.

The clinical presentation, normal neurological examination, unremarkable developmental history, EEG pattern, and unremarkable MRI were most consistent with a GGE phenotype with prominent ECS. The patient was started on levetiracetam at an initial dose of 20 mg/kg/day, which was subsequently titrated to 30 mg/kg/day and then to 40 mg/kg/day based on follow-up clinical assessments and EEG findings. Since treatment initiation in December 2024, she has been seizure-free.

## 3. Discussion

This case demonstrates the unusual co-occurrence of generalized tonic–clonic seizures and ECS in a previously healthy 10-year-old girl. Her clinical presentation, characterized by recurrent unprovoked generalized tonic–clonic seizures, normal neurological examination, and unremarkable brain MRI, supports a probable GGE phenotype. The EEG showed generalized spike-and-wave and polyspike-and-wave discharges at approximately 3 Hz that were triggered by eye closure. However, the posterior maximality of the EEG discharges introduces some electroclinical ambiguity, and the case is therefore best interpreted as a probable GGE phenotype with prominent ECS and general discharges showing posterior predominance, rather than as a fully canonical presentation.

ECS is an EEG abnormality in which epileptiform discharges are triggered by eye closure, but by itself, it is not sufficient to diagnose epilepsy. Epileptiform discharges may occasionally be seen in individuals without a clinical seizure history, especially in the pediatric population [10]. However, when ECS is observed on EEG in a patient with recurrent, unprovoked generalized tonic–clonic seizures, as in this case, it becomes a clinically significant and supportive finding. This phenomenon is thought to occur through a physiological increase in occipital alpha rhythms, reflecting deactivation of the visual cortex. In susceptible patients, this response paradoxically reduces the threshold for generalized spike-wave discharges by enhancing thalamo-cortical synchronization [3].

Most reported ECS cases involve adolescents or adults, with few pediatric descriptions documented. Our patient’s clinical profile also differed from that of typical ECS cohorts. She lacked any history of febrile seizures or familial epilepsy. In contrast, 42% of patients in a previous series by Sevgi and colleagues experienced febrile convulsions during childhood [6]. Additionally, EEM and photosensitivity were absent in our case. In the Sevgi’s series, approximately 40% of patients with ECS demonstrated photic EEG responses. Thus, the combination of a purely eye-closure-sensitive EEG and generalized tonic–clonic seizures with otherwise normal investigations is unusual. Pediatric cases of GGE with ECS without accompanying Jeavons syndrome remain uncommon or underreported. This case suggests that ECS can occur in a pediatric generalized epilepsy phenotype without other reflex signs, thereby adding to the limited literature on this presentation.

In our patient, the EEG findings demonstrated intermittent bursts of generalized spike-and-wave and polyspike-and-wave discharges predominantly triggered by eye closure. These discharges occurred immediately after both spontaneous and instructed eye closure and were more prominent during drowsiness. This pattern is highly consistent with the definition of ECS. Differentiation from benign alpha blocking relies on the paroxysmal morphology and timing relative to closure. Video-EEG confirmed our patient alertness and excluded artifacts or mechanical blink discharges. The absence of photic sensitivity further differentiated ECS from classical photosensitivity.

Fixation-off sensitivity refers to an EEG phenomenon triggered by the elimination of central vision or fixation and may be seen with generalized or posterior-predominant epileptiform discharges [11]. In this patient, the discharges occurred in a well-lit room, suggesting that the activity was linked specifically to eye closure rather than visual deprivation, confirming ECS and excluding fixation-off sensitivity. Because the discharges showed posterior maximality, self-limited occipital epilepsy was considered in the differential diagnosis. Nevertheless, the generalized spike-wave/polyspike-wave morphology, eye-closure dependence, lack of a consistent occipital seizure semiology, and normal MRI favor a generalized epilepsy phenotype with ECS rather than a primary occipital epilepsy syndrome. Moreover, the patient did not exhibit eyelid myoclonic jerks or absences triggered by eye closure, therefore EEM was excluded. However, neither prolonged video-EEG nor sleep EEG was obtained. Given that epileptiform discharges in GGE are often enhanced during drowsiness and sleep [12], these studies might have provided additional electroclinical details and strengthened syndrome classification.

ECS itself does not require a specific therapy beyond the standard GGE management. GGEs are generally quite responsive to broad-spectrum antiseizure medications (ASMs) [1]. Levetiracetam, the medication initiated in our patient, is a broad-spectrum ASM effective against both focal and generalized seizures and is often a first-line choice for generalized epilepsies due to its favorable side effect profile and efficacy [13]. The positive response to levetiracetam, with no further generalized tonic–clonic seizures for approximately 1.5 years, further supports the diagnosis and appropriate management.

The diagnostic significance of ECS in this case is twofold. Firstly, it reinforces the genetic predisposition of epilepsy, as ECS is predominantly observed in GGEs. Secondly, it helps guide treatment and prognosis. Although the patient responded well to levetiracetam, the presence of ECS might influence future treatment decisions or monitoring strategies, especially if other seizure types or symptoms emerge. The fact that epileptiform discharges were more prominent during drowsiness also aligns with the concept that sleep deprivation or drowsiness can exacerbate epileptiform activity in GGEs. Therefore, although eye closure cannot be entirely avoided, clinicians should advise patients with ECS-positive epilepsy to minimize other precipitating factors, such as sleep deprivation.

In addition, this case underscores the importance of careful EEG interpretation, particularly the inclusion of activation procedures like eye closure and opening, to uncover ECS, a subtle but diagnostically significant finding. Although performed under controlled clinical EEG conditions, the same diagnostic principle may be applicable in less controlled or more naturalistic settings, where robust EEG preprocessing and artifact-aware methods are increasingly being developed for real-world use [14]. The absence of clinical correlation with the ECS-induced EEG discharges (i.e., no behavioral arrest or impaired awareness during the EEG events) in our patient is also noteworthy, indicating that these electrographic abnormalities do not always translate into overt clinical seizures. This phenomenon, where subclinical epileptiform discharges are present, is common in various epilepsy syndromes and highlights the distinction between electrographic and clinical seizures.

The prognostic implications of ECS are not fully settled. A prior study conducted on patients with JME revealed that ECS was associated with a more severe disease course [8]; however, a large series found that ECS did not predict poor outcomes [7]. More data are needed, but current evidence suggests ECS in isolation is not necessarily a marker of refractory epilepsy. For instance, in the Sevgi series, most ECS-positive patients (24/25) became seizure-free on treatment [6], aligning with our case.

## 4. Conclusions

This case report describes a child with an uncommon electroclinical picture most consistent with a GGE phenotype with prominent ECS. The findings contribute to the understanding of the various electrophysiological manifestations within GGEs and emphasize the importance of including repeated eye-open/close maneuvers during EEG in pediatric epilepsy to detect ECS. Further research is needed to fully clarify the genetic and pathophysiological mechanisms of ECS in such atypical presentations and its long-term prognostic implications.

## Data Availability

The original contributions presented in this study are included in the article. Further inquiries can be directed to the corresponding author.

## Abbreviations

The following abbreviations are used in this manuscript:

ASM	Antiseizure medication
ECS	Eye-closure sensitivity
EEG	Electroencephalogram
EEM	Eyelid myoclonia
GGE	Genetic generalized epilepsy
JME	Juvenile myoclonic epilepsy
MRI	Magnetic resonance imaging

## References

[1] Verducci C., Friedman D., Donner E., Devinsky O. (2020). Genetic generalized and focal epilepsy prevalence in the North American SUDEP Registry. Neurology.

[2] Montenegro M.A., Valente K. (2024). EEG in focal and generalized epilepsies: Pearls and perils. Epilepsy Behav..

[3] Yalcin A.D., Surmeli R. (2021). Eye closure sensitivity and genetic generalized epilepsies: A prospective study of 123 cases. Epilepsy Res..

[4] Yildirim F., Aydin Z., Sakci Z., Yalcin A.E.D. (2022). Investigation of patients with eye closure sensitive epilepsy with magnetic resonance spectroscopy. Clin. EEG Neurosci..

[5] Zawar I., Toribio M.G.G., Xu X., Alnakhli R.S., Benech D., Valappil A.M.N., Wyllie E., Burgess R., Kotagal P., Lachhwani D. (2022). Epilepsy with Eyelid myoclonias—A diagnosis concealed in other genetic generalized epilepsies with photoparoxysmal response. Epilepsy Res..

[6] Sevgi E.B., Saygi S., Ciger A. (2007). Eye closure sensitivity and epileptic syndromes: A retrospective study of 26 adult cases. Seizure.

[7] Tekin Guveli B., Baykan B., Dortcan N., Bebek N., Gurses C., Gokyigit A. (2013). Eye closure sensitivity in juvenile myoclonic epilepsy and its effect on prognosis. Seizure.

[8] Uchida C.G.P., de Carvalho K.C., Guaranha M.S.B., Guilhoto L., de Araujo Filho G.M., Yacubian E.M.T. (2018). Prognosis of Juvenile myoclonic epilepsy with eye-closure sensitivity. Seizure.

[9] Mermi Dibek D., Baykan B. (2025). Eye closure sensitivity and related EEG findings: Persistence rates and classification of epilepsy syndromes by the International League Against Epilepsy. Epileptic Disord..

[10] Dede H.O., Bebek N., Emekli S., Baykan B., Yapici Z., Gokyigit A. (2021). The clinical significance and electrophysiologic findings of fixation-off and closure of the eyes sensitivity: Data from a prospective unselected population. Epilepsy Res..

[11] Wang X., Zhang Y., Zhang W., Shen C., Jin L., Chen B., Jiang Z., Tao J.X., Liu Y. (2018). The electroclinical features of idiopathic generalized epilepsy patients presenting with fixation-off sensitivity. Epileptic Disord..

[12] Seneviratne U., Cook M.J., D’Souza W.J. (2017). Electroencephalography in the diagnosis of genetic generalized epilepsy syndromes. Front. Neurol..

[13] Hakami T. (2021). Efficacy and tolerability of antiseizure drugs. Ther. Adv. Neurol. Disord..

[14] Ronca V., Flumeri G.D., Giorgi A., Vozzi A., Capotorto R., Germano D., Sciaraffa N., Borghini G., Babiloni F., Arico P. (2024). o-CLEAN: A novel multi-stage algorithm for the ocular artifacts’ correction from EEG data in out-of-the-lab applications. J. Neural Eng..

